# DepLink: an R Shiny app to systematically link genetic and pharmacologic dependencies of cancer

**DOI:** 10.1093/bioadv/vbad076

**Published:** 2023-06-12

**Authors:** Tapsya Nayak, Li-Ju Wang, Michael Ning, Gabriela Rubannelsonkumar, Eric Jin, Siyuan Zheng, Peter J Houghton, Yufei Huang, Yu-Chiao Chiu, Yidong Chen

**Affiliations:** Greehey Children’s Cancer Research Institute, University of Texas Health San Antonio, San Antonio, TX 78229, USA; UPMC Hillman Cancer Center, University of Pittsburgh, Pittsburgh, PA 15232, USA; Greehey Children’s Cancer Research Institute, University of Texas Health San Antonio, San Antonio, TX 78229, USA; UPMC Hillman Cancer Center, University of Pittsburgh, Pittsburgh, PA 15232, USA; Greehey Children’s Cancer Research Institute, University of Texas Health San Antonio, San Antonio, TX 78229, USA; Greehey Children’s Cancer Research Institute, University of Texas Health San Antonio, San Antonio, TX 78229, USA; Greehey Children’s Cancer Research Institute, University of Texas Health San Antonio, San Antonio, TX 78229, USA; Department of Population Health Sciences, University of Texas Health San Antonio, San Antonio, TX 78229, USA; Greehey Children’s Cancer Research Institute, University of Texas Health San Antonio, San Antonio, TX 78229, USA; Department of Molecular Medicine, University of Texas Health San Antonio, San Antonio, TX 78229, USA; UPMC Hillman Cancer Center, University of Pittsburgh, Pittsburgh, PA 15232, USA; Division of Hematology/Oncology, Department of Medicine, University of Pittsburgh, Pittsburgh, PA 15261, USA; UPMC Hillman Cancer Center, University of Pittsburgh, Pittsburgh, PA 15232, USA; Division of Hematology/Oncology, Department of Medicine, University of Pittsburgh, Pittsburgh, PA 15261, USA; Greehey Children’s Cancer Research Institute, University of Texas Health San Antonio, San Antonio, TX 78229, USA; Department of Population Health Sciences, University of Texas Health San Antonio, San Antonio, TX 78229, USA

## Abstract

**Motivation:**

Large-scale genetic and pharmacologic dependency maps are generated to reveal genetic vulnerabilities and drug sensitivities of cancer. However, user-friendly software is needed to systematically link such maps.

**Results:**

Here, we present DepLink, a web server to identify genetic and pharmacologic perturbations that induce similar effects on cell viability or molecular changes. DepLink integrates heterogeneous datasets of genome-wide CRISPR loss-of-function screens, high-throughput pharmacologic screens and gene expression signatures of perturbations. The datasets are systematically connected by four complementary modules tailored for different query scenarios. It allows users to search for potential inhibitors that target a gene (Module 1) or multiple genes (Module 2), mechanisms of action of a known drug (Module 3) and drugs with similar biochemical features to an investigational compound (Module 4). We performed a validation analysis to confirm the capability of our tool to link the effects of drug treatments to knockouts of the drug’s annotated target genes. By querying with a demonstrating example of *CDK6*, the tool identified well-studied inhibitor drugs, novel synergistic gene and drug partners and insights into an investigational drug. In summary, DepLink enables easy navigation, visualization and linkage of rapidly evolving cancer dependency maps.

**Availability and implementation:**

The DepLink web server, demonstrating examples and detailed user manual are available at https://shiny.crc.pitt.edu/deplink/.

**Supplementary information:**

Supplementary data are available at *Bioinformatics Advances* online.

## 1 Introduction

With revolutionizing techniques in high-throughput screening and genomic profiling, large-scale cancer dependency maps, also known as the DepMaps, are rapidly growing, providing insights into genetic vulnerabilities and pharmacologic sensitivities of pan-cancer cell lines. These dependency maps are generated by observing the changes in cancer cell viability induced by systematically knocking out genes using genome-wide CRISPR screens (Behan *et al.*, 2019; Dempster *et al.*, 2021) or by treating cells with small compounds using multiplexed drug screens (Corsello *et al.*, 2020; Iorio *et al.*, 2016). Focused on the molecular effects of treatments, the NIH Library of Integrated Network-based Cellular Signatures (LINCS) Consortium generated ‘molecular signatures’ of perturbations, i.e. changes in gene expression profiles induced by a treatment, at an unprecedented scale (Subramanian *et al.*, 2017). These projects play a significant role in propelling precision oncology by identifying and targeting the ‘Achilles heel’ of cancer (Vazquez and Sellers, 2021). Several interactive web servers have been developed to make these data resources more accessible to broad cancer research community. For example, shinyDepMap is a web browser that visualizes gene clusters with similar dependency profiles (Shimada *et al.*, 2021). The Data Explorer tool accompanying the DepMap data of the Broad Institute visualizes the correlation between two user-selected genes or compounds. However, current tools do not support systematic analyses across all genes or drugs. It also remains lacking user-friendly software, which meaningfully links and visualizes heterogeneous screening and molecular datasets to investigate similar mechanisms behind perturbations triggered by genes or compounds. To address this issue, here, we present the DepLink web application to answer two important questions in cancer biology:

- Which drugs can be potential surrogates to knockout of a gene in cancer cells?
- Which genes are potential targets or mechanisms of action of a drug?

DepLink is an R Shiny app designed to integrate large-scale data of genetic and drug screens, molecular signatures and cheminformatics. The goal is to enable researchers to identify genetic (gene knockouts) and pharmacologic perturbations (drug treatments) that induce similar effects in terms of cell viability or molecular changes. DepLink has four interconnected modules that allow bi-directional query scenarios from a gene or multiple genes to a drug, or from a known drug or new compound to a gene. Query results are presented via a user-friendly interface of interactive networks and figures, as well as easy-to-operate data tables, enabling meaningful analyses with no requirement of programming skills.

## 2 Methods

**Figure 1.**
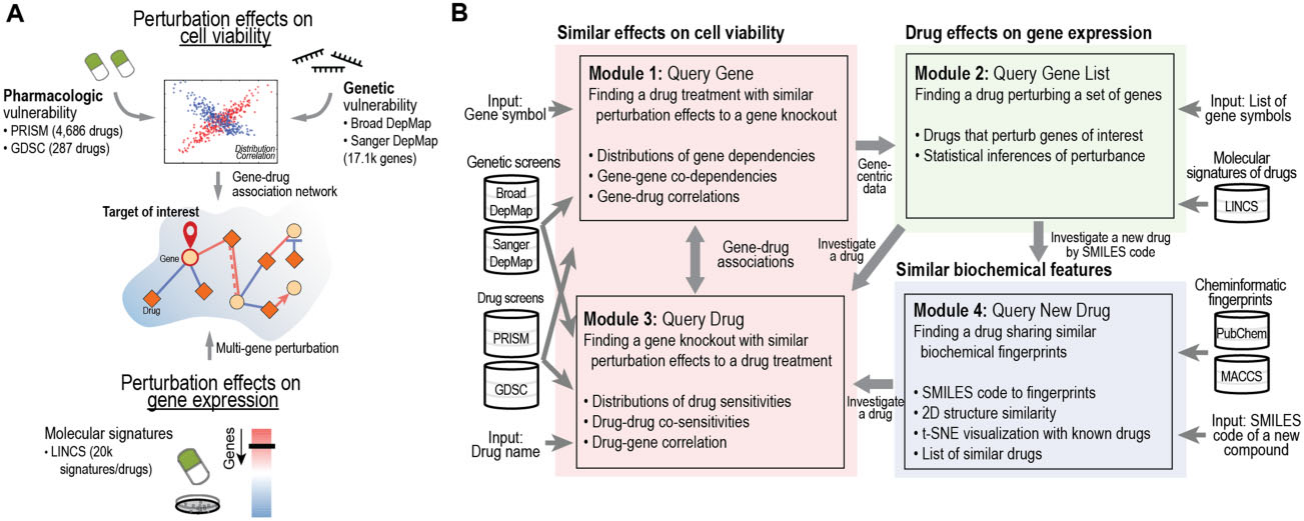
Overview of DepLink. (**A**) Graphic abstract of DepLink. DepLink is an R Shiny app designed to answer two important questions: (i) which drugs can be potential surrogates to knockout of a gene in cancer cells, and (ii) which genes are potential targets or mechanisms of action of a drug. (**B**) Schematic overview of DepLink. DepLink allows the identification of similar (or opposite) effects induced by genetic and pharmacologic perturbations in cell viability (highlighted in red) and gene expression (green). It systematically incorporates (i) two genome-wide CRISPR loss-of-function screens [Broad and Sanger Cancer Dependency Map (DepMap) projects], (ii) high-throughput pharmacologic screens (PRISM and GDSC) and (iii) a next-generation Connectivity Map of molecular signatures (LINCS). DepLink also allows a user to investigate a new drug of interest based on its biochemical features (highlighted in indigo). MACCS, Molecular ACCess System; SMILES, Simplified Molecular Input Line Entry System

As shown in Figure 1A, DepLink integrates heterogeneous screening and molecular datasets of three categories: (i) genome-wide CRISPR loss-of-function screens: Broad and Sanger DepMap projects, each covering genome-wide gene knockouts (Behan *et al.*, 2019; Dempster *et al.*, 2021); (ii) high-throughput drug screens: Profiling Relative Inhibition Simultaneously in Mixtures (PRISM; primary screen) (Corsello *et al.*, 2020) and Genomics of Drug Sensitivity in Cancer (GDSC) (Iorio *et al.*, 2016); and (iii) molecular signatures of perturbations: The LINCS collection of molecular (gene expression) signatures induced by drug treatments (Subramanian *et al.*, 2017). Four complementary and interconnected modules are designed in DepLink to integrate the datasets for different query needs (Fig. 1B):

- Modules ‘1. Query Gene’ and ‘3. Query Drug’ take a gene or drug as input and search for perturbations with similar inhibitory effects on cell viability across pan-cancer cell lines or in a specific cancer type. See Supplementary Methods for details on data sources and preprocessing methods used in this module.
- Module ‘2. Query Gene List’ identifies a drug that perturbs the list of target genes by searching among the molecular signatures of LINCS. Details on the computation of statistical significance of drugs that perturb a set of genes are described in Supplementary Methods.
- Module ‘4. Query New Drug’ converts a canonical Simplified Molecular Input Line Entry System (SMILES) code of a novel compound to biochemical fingerprints, and searches for similar drugs based on Tanimoto similarity (*S*_Tanimoto_; Supplementary Methods).

DepLink was implemented using R (version 4.0.1) and R shiny server (version 1.5.14.948). It uses several R libraries of data processing and visualization, including Plotly, Tidyverse, visNetwork and Venn Diagram.

## 3 Results

### 3.1 The user interface of DepLink

Figure 1B summarizes the goal and input/output of each query module, and connections among the modules. The user interface is described in Supplementary Methods and shown in Supplementary Figure S1.

### 3.2 Global correlation network of gene knockouts and drugs

We started by building and examining a global correlation network among all gene knockouts and drugs. Gene–drug, gene–gene and drug–drug correlation coefficients were calculated based on 456 common cell lines between the Broad DepMap (Chronos gene-effect scores that infer gene fitness effects) and PRISM (log-fold changes in cell counts associated with drug treatments) datasets. We set cutoffs on correlation coefficients to yield a visualizable size of network with the largest average node degree of 6.3, comprising 123 genes, 396 drugs and 1630 edges (cutoffs, 0.61, 0.61 and 0.27 on gene–gene, drug–drug and gene–drug correlations; see Supplementary Fig. S2 and Supplementary Methods). The network contained clusters of drugs that were designed to inhibit important oncogenic and cancer progression pathways, such as MEK inhibitors (e.g. selumetinib and cobimetinib) with genes in the BRAF-MAPK pathway (e.g. *BRAF*, *MAPK1* and *MAP2K1*) and inhibitors of the ErbB family (gefitinib, osimertinib and dacomitinib) (Supplementary Fig. S3). Genes involved in the mitochondrial respiratory chain formed a dense cluster that included cytochrome c oxidase genes (*COX5B*, *COX6B* and *COX7C*) and NADH: ubiquinone enzymes (*NDUFA2*, *NDUFS1* and *NDUFS2*). *MDM2* inhibitors (nutlin-3 and idasanutlin) formed a cluster with many DNA repair genes that govern cell apoptosis (*TP53*, *CHEK2*, *ATM* and *MDM2*). Two key players of the cell cycle, *CDK6* and *CCND1*, were connected to palbociclib, the first CDK4/6 inhibitor approved for cancer therapy. The results are available under the ‘Global Network’ tab of DepLink.

### 3.3 Validation analysis using annotated drug targets

To validate the correlation analysis implemented in DepLink, we analyzed 11 008 drug–target gene annotations collected from PRISM, comprising 3464 drugs and 1796 target genes. Broad DepMap gene-effect scores of the target genes were available in 98.2% (10 811) pairs that corresponded to 3443 drugs. Treatment responses to 774 (22.5%) drugs were significantly correlated with gene-effect scores of at least one intended target of the drug (with Pearson correlation *P* < 0.05). We also calculated an observed-to-expected (O/E) ratio of annotated drug–target pairs passing a threshold on gene–drug correlation coefficients (see Supplementary Methods). All thresholds we tested (0–0.7) yielded statistically significant results (right-tailed Fisher’s exact test *P*-values <0.05; Supplementary Fig. S4). The cutoff of 0.27 on gene–drug correlation used to construct the global network achieved a significant O/E ratio (512.0; *P* = 5.7 × 10^−174^). Phase 3 drugs demonstrated an even more evident O/E ratio of 1555.9 (*P* = 2.1 × 10^−40^). Thus, our validation analysis demonstrated the capability of DepLink in recapitulating known drug–target gene relationships.

### 3.4 Use case—identification of drugs and genes associated with *CDK6* knockout

**Figure 2.**
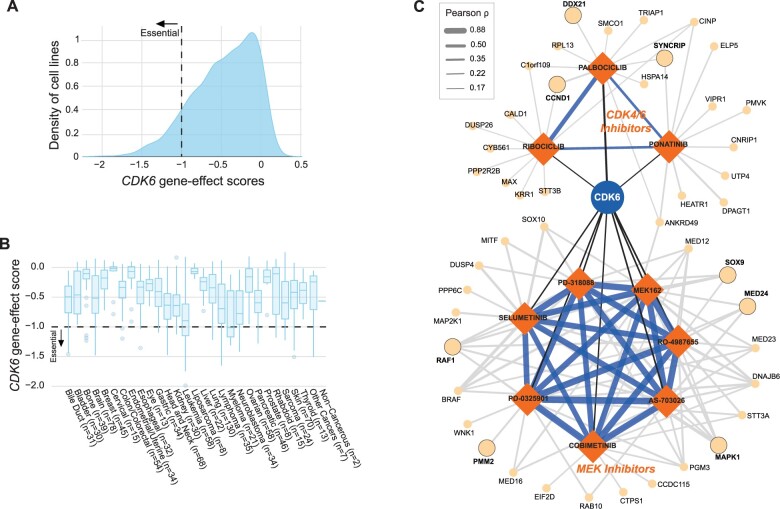
Investigations of *CDK6* dependencies, CDK4/6 inhibitors and potential synergetic drug partners. (**A**) Pan-cancer distribution of Chronos gene-effect score of *CDK6* across 1054 cancer cell lines in the Broad DepMap dataset. A greater negative score indicates a stronger dependency on *CDK6*. Dashed line denotes the cutoff of −1 for identifying *CDK6* as an essential gene in a cell line. (**B**) Distribution of *CDK6* gene-effect scores across 27 cancer lineages and 2 other groups. (**C**) Correlation network of *CDK6*. We calculated pairwise correlation coefficients between a gene knockout (gene-effect scores from Broad DepMap) and a drug response (log-fold changes from PRISM). With *CDK6* as the hub gene, the network was constructed by the 10 drugs with the strongest correlation coefficients to the *CDK6* knockout. To further illuminate potential mechanisms of action, for each drug, we identified the top 10 correlated gene knockouts and incorporated them into the network. Color and width of edges denote the direction and magnitude of correlation, respectively. The gene names in bold correspond to significant molecular biological processes (*P* < 0.05) corresponding to G2-M Checkpoint, Myc Targets V1 and Notch signaling for CDK6 inhibitors and Glycolysis and PI3K/AKT/mTOR signaling for MEK inhibitors. See Supplementary Methods for more details on network construction and visualization

As a use case of DepLink, we further investigated *CDK6*, as it is one of the most promising targets of cell cycle-based cancer therapy. Here, we summarized results from Modules 1–3 and focused our analyses on the Broad DepMap and PRISM screens to study similarities in cell viability inhibition induced by *CDK6* knockout, other gene knockouts, CDK4/6 inhibitors and other drugs. *CDK6* was found to be an essential gene in 109 of the 1054 cell lines assayed by the Broad DepMap (Fig. 2A). In particular, viability of myeloma, leukemia and neuroblastoma cells was strongly dependent on *CDK6* (median scores, −1.02, −0.90 and −0.78; Fig. 2B). We calculated a Pearson correlation coefficient between the gene-effect scores of *CDK6* and log-fold changes of cell viability induced by each drug assayed by PRISM across 456 common cell lines. Two clusters were formed among the top 10 drugs achieving similar effects with *CDK6* knockout (Fig. 2C and Supplementary Methods). One included two CDK4/6 inhibitors, palbociclib and ribociclib (*ρ* = 0.30 and 0.18), as well as ponatinib (which promotes G1 cell cycle arrest and synergizes with palbociclib) (Liu *et al.*, 2019; Schneeweiss-Gleixner *et al.*, 2019). Palbociclib and ribociclib showed highly similar effects in cell viability (*ρ* = 0.58 across 568 cell lines; Supplementary Fig. S5A), well beyond many other known *CDK4* and/or *CDK6* inhibitors (Supplementary Fig. S5B).

The other cluster of drugs correlated with *CDK6* knockout was composed of seven MEK inhibitors (Fig. 2C). Cobimetinib is FDA-approved to treat *BRAF*-mutated metastatic melanoma in combination with vemurafenib. Among all types of cancer, skin cancer cells responded best to cobimetinib (median of log-fold changes in viability = −1.93 among 43 cell lines; Supplementary Fig. S5C). The seven MEK inhibitors formed an interconnected network (Fig. 2C). All were among the top 10 highly correlated drugs of cobimetinib (Supplementary Table S1), with correlation coefficients ranging between 0.88 (PD-0325901) and 0.69 (selumetinib). Our data revealed potential synergistic effects between CDK and MEK inhibitors (see Supplementary Discussion).

### 3.5 Use case—examination of a novel inhibitor of *CDK6*

The ‘Query New Drug’ module of DepLink allows users to investigate a novel drug by a SMILES code and analyze its proximity to known drugs based on molecular fingerprints. As an example, we analyzed an investigational CDK4/6 inhibitor in Phases 2 and 3 clinical trials, namely SHR6390, with its canonical SMILES code. DepLink calculated Tanimoto similarities (*S*_Tanimoto_) between SHR6390 and all drugs screened by PRISM based on 1047 binary-state biochemical features of PubChem and MACCS. Palbociclib and ribociclib were the top and third most similar drugs (*S*_Tanimoto_=0.96 and 0.82, respectively). Palbociclib and SHR6390 shared 237 common biochemical features out of 244 and 239 features present in palbociclib and SHR6390, respectively (Supplementary Fig. S6). TGX-221, a PI3K inhibitor targeting *PIK3CB* and *PIK3CD*, was the second most similar drug to SHR6390 (*S*_Tanimoto_, 0.82).

## 4 Conclusion

Large-scale genetic and pharmacologic dependency maps demand a platform where one can navigate, visualize and link information across these complex datasets. DepLink is, as far as we know, the first a user-friendly analysis workflow designed to address the research gap by querying across a gene or multiple genes, and a drug. Moreover, it enables researchers to analyze biochemical features of a new compound and to study its potential mechanisms of action through known drugs with similar biochemical features. We expect DepLink to continuously evolve with rapidly growing high-throughput screening data resources.

## Supplementary Material

vbad076_Supplementary_Data

## Data Availability

The data utilized in this study are sourced from their respective original repositories as detailed in the Methods and Supplementary Methods sections. The analyzed data can be accessed through the DepLink website at https://shiny.crc.pitt.edu/deplink/.
