# Supplementary material for: DepLink: an R Shiny app to systematically link genetic and pharmacologic dependencies of cancer
Source: Bioinform Adv. 2023 Jun 12;3(1):vbad076. doi: 10.1093/bioadv/vbad076 (PMC10290235; doi:10.1093/bioadv/vbad076)
Supplement: vbad076_Supplementary_Data [file vbad076_supplementary_data.pdf]

Supplementary Information

DepLink: an R Shiny app to systematically link genetic and pharmacologic dependencies of cancer

Tapsya Nayak[1,†], Li-Ju Wang[2,†], Michael Ning[1,2], Gabriela Rubannelsonkumar[1], Eric Jin[1], Siyuan Zheng[1,3], Peter J. Houghton[1,4], Yufei Huang[2,5], Yu-Chiao Chiu[2,5*], Yidong Chen[1,3*]

[1]Greehey Children's Cancer Research Institute, University of Texas Health San Antonio, San Antonio, TX 78229, USA, [2]UPMC Hillman Cancer Center, University of Pittsburgh, Pittsburgh, PA 15232, USA, [3]Department of Population Health Sciences, University of Texas Health San Antonio, San Antonio, TX 78229, USA, [4]Department of Molecular Medicine, University of Texas Health San Antonio, San Antonio, TX 78229, USA, [5]Division of Hematology/Oncology, Department of Medicine, University of Pittsburgh, Pittsburgh, PA 15261, USA.

Contents

Supplementary Methods 3
- Data sources and pre-processing 3
- DepLink user interface 3
- Construction of a global correlation network among gene knockouts and drugs 4
- Similarity analysis among gene knockouts and drug treatments 4
- Statistical inference of drugs that perturb a set of genes (Module "Query Gene List") 4
- Validation analysis using observed-to-expected (O/E) ratio of annotated drug targets 5
- Similarity analysis of biochemical fingerprints for a new query drug 5
- Generating the visualization of the *CDK6* network shown in Fig. 2C of main text 6

Supplementary Discussion 7

Supplementary Table 8
- Supplementary Table S1. 8

Supplementary Figures 9
- Supplementary Fig. S1. 9
- Supplementary Fig. S2. 10
- Supplementary Fig. S3. 11
- Supplementary Fig. S4. 12
- Supplementary Fig. S5. 13
- Supplementary Fig. S6. 14

Supplementary References 15

Supplementary Methods

Data sources and pre-processing

For the two CRISPR loss-of-function screens of the Broad (version 21Q4) and Sanger (21Q2) DepMap projects, we used Chronos-normalized gene-effect scores to measure gene fitness effects (Behan, et al., 2019; Dharia, et al., 2021). A more negative gene-effect score indicates stronger essentiality of the gene in a cell line, and a score < -1 denotes effective inhibition of cell viability by knocking out the gene. All downloaded data were pre-processed to remove any cell line IDs labeled as "FAIL" or blank (1,054 and 318 cell lines, respectively). Gene dependency data were processed to extract only genes shared in the Broad DepMap and Sanger CRISPR datasets (17,078 common genes) (Dempster, et al., 2019).

For drug screens, PRISM measures treatment response by log-fold changes in cell counts relative to DMSO (4,686 compounds and 568 cell lines in version 19Q4). GDSC uses the half-maximal inhibitory concentration (IC_{50}). The downloaded GDSC data set combined drugs from GDSC1 and GDSC2; this was reorganized to extract an IC_{50} value per cell line/drug pair. For duplicate drugs per cell line, only one value (first occurrence) was chosen for representational purposes (287 compounds and 973 cell lines in version 2019). A total of 205 drugs were shared between PRISM and GDSC (Corsello, et al., 2020). All screening datasets were downloaded from the Data Portal of Broad DepMap (https://depmap.org/portal/download).

We analyzed the molecular signatures of LINCS to determine if a list of query genes is up- or down- regulated by a drug. The dataset was downloaded from the LINCS database (GSE92742), which contains changes in expression levels of 12,328 genes induced by treatment of 19,811 drugs. We used level-5 data of *Z*-scores computed by comparing inferred $\log_2$-RPKM values between post- and pre-treatment samples for each cell line at each dose and time point, summarized by a weighted average across replicates. For a cell line-drug pair with multiple doses and/or time points, we use the one with the largest standard deviation as the representative sample as it achieves the strongest response after treatment. A series of statistical tests are performed by DepLink to infer whether the query list of genes is significantly perturbed by each drug in the LINCS dataset.

DepLink user interface

Supplementary Figure S1 shows the homepage of DepLink. The navigation bar as a top ribbon of the webpage allows users to select a function, including viewing the global network among gene knockouts and drugs, four main modules for querying gene(s) or a known/novel drug, and a "Help" section. In each module, user starts a query by selecting from a dropdown list for gene names, drug names, and/or cancer types, or typing in a text box. For each input item, a pre-filled default query input or an example is provided to assist users and/or clarify the input requirement. The help page provides detailed documentation of the overall tool and each module, including data sources and examples. Every output figure or table has a question mark button that provides a description and/or legends.

Construction of a global correlation network among gene knockouts and drugs

The main goal of DepLink is to link CRISPR and drug screens to identify similar inhibitory effects on cell viability between a gene knockout and a drug treatment. Pearson correlation coefficients were computed between gene-effect scores (similarities between two gene knockouts), between gene-effect scores and drug responses (between a knockout and a drug treatment), or between drug responses. We implemented the Pearson correlation as the measure of similarity because the interpretation of results is straightforward and meaningful for most biomedical researchers. Thus, the PRISM study performed correlation analysis on data of gene knockouts and drug screens to validate a drug's intended targets (Corsello, et al., 2020). We constructed a global correlation network by pairwise analysis among all genes from Broad DepMap and all drugs in PRISM on 456 common cell lines between the two datasets. We performed a systematic analysis to determine the optimal cutoffs on correlation coefficients for constructing the global gene-drug correlation network. To ensure that the resulting network size is manageable and can be easily visualized, we identified correlation thresholds that yielded 100-1000 connections in each category. For gene-gene connections, thresholds on absolute correlation coefficients >0.40 and >0.66 yielded 1000 and 100 gene-gene connections, respectively (Supplementary Fig. S2). Similarly, we identified cutoffs of 0.61 and 0.77 for drug-drug pairs, and 0.23 and 0.36 for gene-drug pairs. Within these ranges, we analyzed all networks constructed by any possible combinations of cutoffs with a step size of 0.01. As a result, the thresholds of 0.61, 0.61, 0.27 on gene-gene, drug-drug, and gene-drug correlations, respectively, were selected as they yielded a network with the largest average degree of 6.3 (123 genes, 396 drugs, and 1,630 edges; Supplementary Fig. S3), indicating a dense network. While using the DepLink tool, users can specify different criteria using slide bars. Pairs meeting the criteria are merged in a network with nodes representing genes and drugs, and edges denoting correlations.

Similarity analysis among gene knockouts and drug treatments

In main query modules ("Query Gene" and "Query Drug"), we generated networks with "second-degree" connections to visualize the top 10 first-degree correlations of the queried gene or drug, and second-degree correlations among these top 10 nodes. So, if a user begins with a query gene to find the ten most highly correlated drugs (first-degree connection) with respect to cell viability. Among those ten drugs, a second-degree edge is established between two drugs if the correlation is ranked among the top 10 for either or both drugs under examination.

Statistical inference of drugs that perturb a set of genes (Module "Query Gene List")

We analyzed the molecular signatures of LINCS to determine if a list of query genes ($\boldsymbol{G}_{query}$) is up- or down- regulated by a drug. Mathematically, the *Z*-score of gene *g* following treatment of drug d in cell line c can be represented as $\boldsymbol{Z}_d(g,c)$, where $c \in [1..C_d]$, $d \in [1..D]$, C_d is the number of cell lines tested for drug *d*, and D denotes the number of drugs. Three statistical tests were calculated to infer the significance of changes in gene expression due to drug treatment *d*:

- **Per-gene *P*-value:** For a gene g, a one-tailed *t*-test was performed to calculate *Z*-scores across cell lines, $\{\boldsymbol{Z}_d(g,c)|c \in [1..C_d]\}$, against zero to ensure the drug significantly up- (or down-) regulates the gene across different cells.
- **Across-gene *P*-value:** An average *Z*-score was calculated per gene across cell lines to measure the overall change in gene expression levels by a drug, *i.e.*, $Z_d^g = \overline{\{\boldsymbol{Z}_d(g,c)|c \in [1..C_d]\}}$. We then applied a one-tailed *t*-test to the average *Z*-scores across the query genes, $\{Z_d^g | g \in \boldsymbol{G_{query}}\}$, against zero. The test is designed to ensure a drug achieves significant effects across the query genes.
- **Permutation *P*-value:** We performed a permutation test to assess the degree of unintended perturbance by a drug. That is, we tested the degree to which $\overline{\{Z_d^g | g \in \boldsymbol{G_{query}}\}}$ is stronger than the same term calculated from randomly selected genes $\overline{\{Z_d^g | g \in \boldsymbol{G_{random}}\}}$, where $\boldsymbol{G_{random}}$ is a set of randomly selected genes with the same size as $\boldsymbol{G_{query}}$. In each iteration of permutation, we randomly selected $|\boldsymbol{G_{query}}|$ genes from all genes and calculated the mean of averaged Z-scores among the selected genes. We then built a normal distribution based on the means and standard deviations of the 100,000 permutations and used the distribution to assess the empirical *P*-value of the observed result. Like the previous two tests, the permutation test was performed with a one-tailed setting based on a user's selection of up- or down-regulation of query genes.

Validation analysis using observed-to-expected (O/E) ratio of annotated drug targets

We validated the results using drug target annotations provided by the PRISM project. For each annotated gene-drug pair, we calculated the correlation coefficient using corresponding data from PRISM and Broad DepMap. Multiple targets of a drug were analyzed independently. We calculated an O/E ratio between the observed number of annotated gene-drug pairs passing a threshold on absolute correlation coefficients (0 to 0.7 with a step of 0.01) versus an expected number estimated by the product of the number of all gene-drug pairs passing a threshold and the fraction of annotated gene-drug pairs among all gene-drug combinations. Statistical significance of an O/E ratio was assessed by the right-tailed Fisher's exact test.

Similarity analysis of biochemical fingerprints for a new query drug

Drug fingerprints that consist of sequences of binary bits are commonly used to characterize molecular compounds or to find similarities. We generated drug fingerprints from all drugs of PRISM using canonical Simplified Molecular Input Line Entry System (SMILES) codes by the R/rcdk package (Guha, 2007). In DepLink, a drug fingerprint contains 881 bits defined by PubChem and 166 bits by the Molecular ACCess System (MACCS) keys. The Tanimoto similarity index is used to assess the similarity between a query drug and any drug included in PRISM,

$$S_{Tanimoto}(F_a, F_b) = \frac{F_a \cap F_b}{F_a \cup F_b}$$

, where F_a and F_b are fingerprints of drugs *a* and *b*, respectively. Dimension reduction on molecular drug fingerprints using *t*-SNE enables visual inspection of relative distances among the query drug and its top similar drugs in the database.

Generating the visualization of the *CDK6* network shown in Fig. 2C of main text

The *CDK6* network shown in Fig. 2C of main text was generated by combining correlation networks from modules 'Query Gene' and 'Query Drug' to help visualize the validation of CDK4/6 network and serve as an example on how users can use DepLink. To generate the network using DepLink, one should search 'PRISM' from the output table (Panel 1H) under the module 'Query Gene', sort the column 'Correlation with Broad DepMap', and the *CDK6* network is generated in Panel 1I. Similarly, the top 10 genes from each of the CDK4/6 and MEK inhibitors from Fig. 2C of main text can be visualized from the DepLink by entering the drug name in the 'Query Drug' module and select the PRISM database. Upon sorting the column 'Correlation with Broad DepMap', the top 10 genes are selected from Panel 3H of the web tool, excluding *CDK6*. If any two or more genes exhibited the same correlation with a query drug, either one of them was selected. The data table was exported to Cytoscape for better network visualization. Finally, the network was slightly modified using Adobe Illustrator for publication purposes.

Supplementary Discussion

By querying with *CDK6*, we demonstrated the capability of DepLink to identify well-characterized gene-drug relationships and shed light on synergistic effects between CDK and MEK inhibitors and investigational CDK4/6 inhibitors. Analysis of drugs highly correlated to *CDK6* knockout revealed two distinct clusters: well-known CDK4/6 inhibitors and novel MEK inhibitors. Our data revealed potential synergistic anti-tumor effects between the two types of inhibitor drugs, both of which are designed to inhibit cell proliferation while action on different pathways. MEK inhibitors target the MAPK pathway, which is responsible for regulating expression levels of D-type cyclins; these in turn regulate cell cycles. Cyclin D regulates *CDK4* and *CDK6* activation, and therefore plays a crucial role in various cancer types (Lavoie, et al., 1996; Scheiblecker, et al., 2020). Both *CDK4* and *CDK6* are part of the G1 cell cycle checkpoint and allow cells to enter S-phase (Meyerson and Harlow, 1994). In BRAF-V600E-mutated melanomas that are refractory to BRAF/MEK inhibitors, a combination therapy of CDK4/6 (palbociclib) and MEK (trametinib) inhibitors significantly suppressed cell-cycle pathways and reduced tumor growth both in vivo and in vitro (Nassar, et al., 2021). This demonstrating example shows the capability of DepLink in not only identifying well-known drugs but also shedding light on novel mechanisms that warrant further investigations.

Supplementary Table

Supplementary Table S1.

Top 10 similar drugs to cobimetinib with respect to treatment response.

Drug name	Broad ID	Correlation coefficient with cobimetinib	Dose
**PD-0325901**	BRD-K49865102-001-08-4	0.883	2.5
**AZD8330**	BRD-K37687095-001-06-9	0.859	2.5
**TAK-733**	BRD-K26667523-001-02-5	0.850	2.5
**Trametinib**	BRD-K12343256-001-08-9	0.849	2.5
**Ro-4987655**	BRD-K64538373-001-01-4	0.838	2.5
**AS-703026**	BRD-K89014967-001-04-3	0.829	2.5
**BVD-523**	BRD-K28965160-001-01-1	0.755	2.5
**MEK162**	BRD-K82244583-001-01-3	0.732	2.5
**PD-318088**	BRD-A57798112-001-03-7	0.725	2.5
**Selumetinib**	BRD-K57080016-001-15-9	0.690	2.5

Supplementary Figures

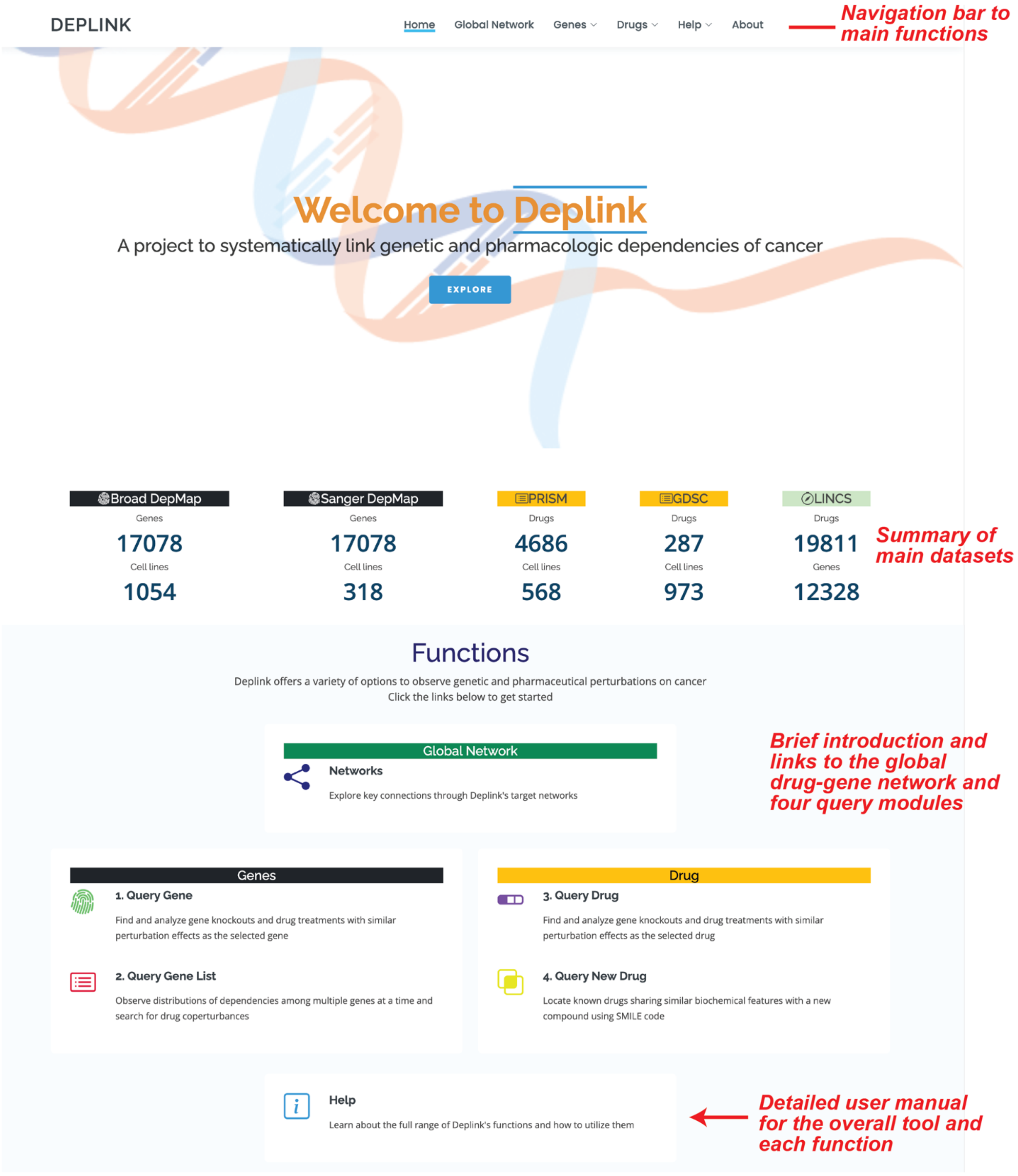

Supplementary Fig. S1.
Screenshot of the homepage of DepLink and descriptions of each section (red font). The tool is available at https://shiny.crc.pitt.edu/deplink/.

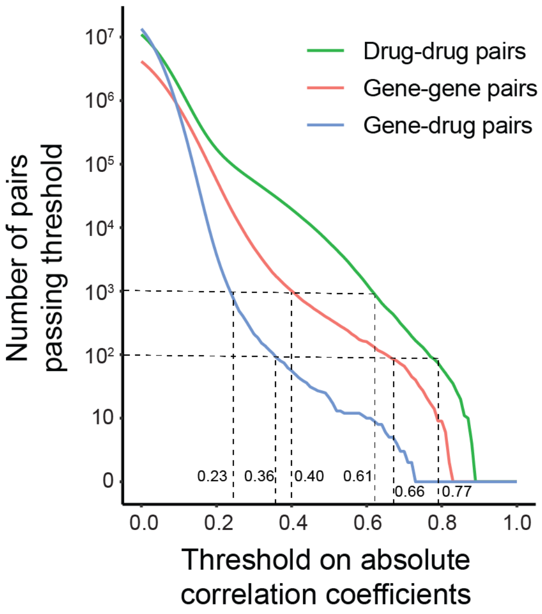

Supplementary Fig. S2.

Number of drug-drug, gene-gene, and gene-drug pairs with absolute correlation coefficients greater than a threshold. Dashed lines denote the thresholds that yielded 100 and 1,000 connections in each category. We then searched within the ranges for optimal cutoffs.

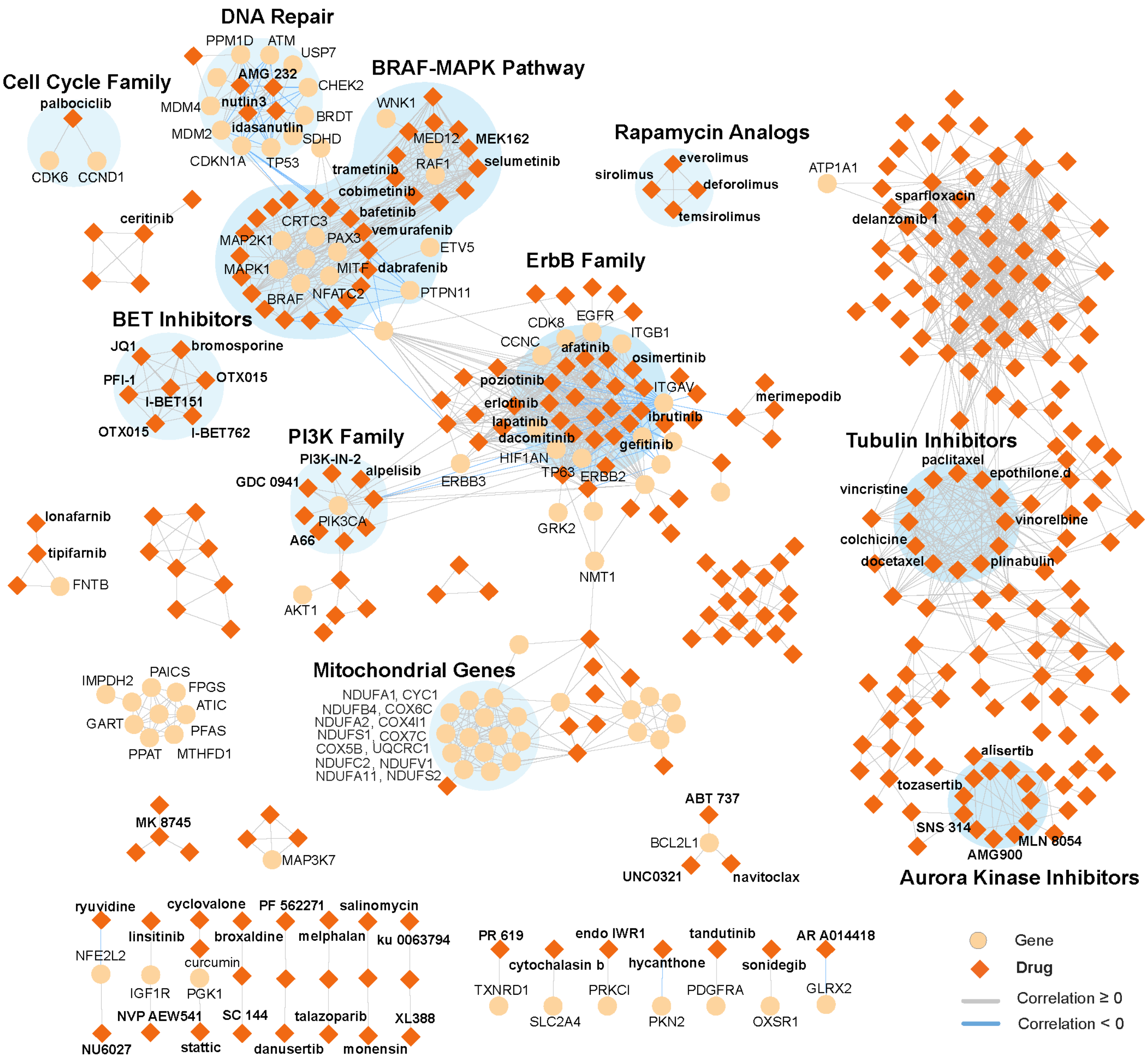

Supplementary Fig. S3.

Global correlation network of gene knockouts and drug treatments. The network was constructed based on the Broad DepMap and PRISM screens. Pearson correlation coefficients were calculated between two genes (gene-effect scores), two drugs (log-fold changes), and a gene and a drug using common cell lines shared between the two datasets. To identify top correlated pairs in each group, correlation coefficients were set at 0.61 for gene-gene and drug-drug pairs and 0.27 for gene-drug pairs. In the network, edges and nodes represent genes/drugs and highly correlated pairs, respectively. Color of an edge denotes the direction (i.e., sign) of correlation. The network is available interactively in the "Global Network" tab of DepLink with options to specify different criteria on correlation coefficients and filter for genes/drugs and clusters. The figure nodes, edges and cluster locations, characteristics, and labels were adjusted and/or added using the Cytoscape software. The Cytoscape (.cys) file of this figure, as well as a text file of all edges, can be downloaded from the DepLink website under the 'Help' tab. Lastly, the figure was prepared using Adobe Illustrator to ensure visual quality for publication.

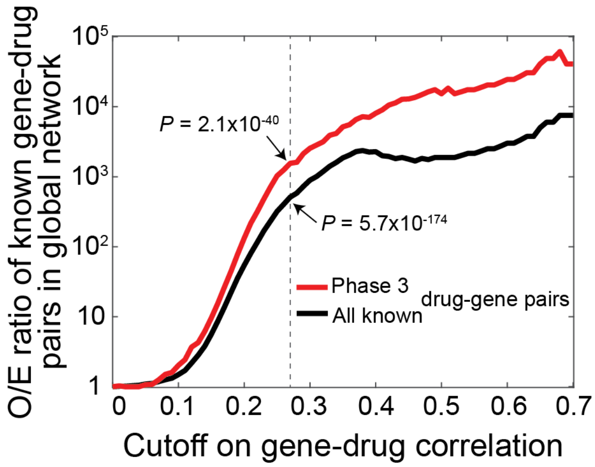

Supplementary Fig. S4.

Observed-to-Expected (O/E) ratio of annotated drug-gene pairs captured in the global correlation network by different thresholds on gene-drug correlation coefficients. Annotation of drug targets was collected from the PRISM database. Drugs with the "phase 3" label were highlighted in red for their clinical value. An O/E ratio was calculated between the observed number of annotated gene-drug pairs passing a threshold on absolute correlation coefficients (0 to 0.7 with a step of 0.01) versus an expected number estimated by the product of the number of all gene-drug pairs passing a threshold and the fraction of annotated gene-drug pairs among all gene-drug combinations. Statistical significance of overrepresentation was assessed by the right-tailed Fisher's exact test. Dashed line denotes the cutoff of 0.27 used to generate the global correlation network shown in Supplementary Fig. S3.

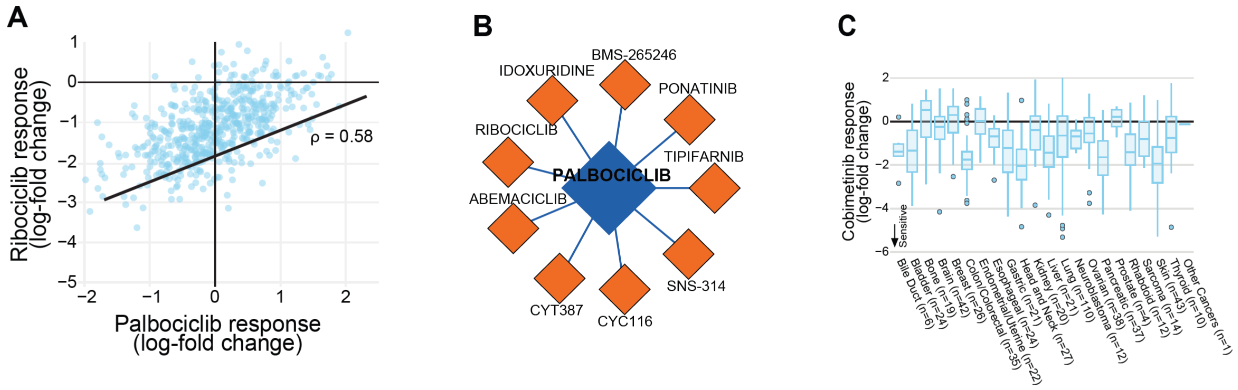

Supplementary Fig. S5.

(A) Correlation in treatment responses between two *CDK6* inhibitors, palbociclib and ribociclib, in the PRISM screen. (B) Network of the 10 drugs whose responses were most correlated to response to palbociclib. (C) Treatment responses to cobimetinib across cancer lineages.

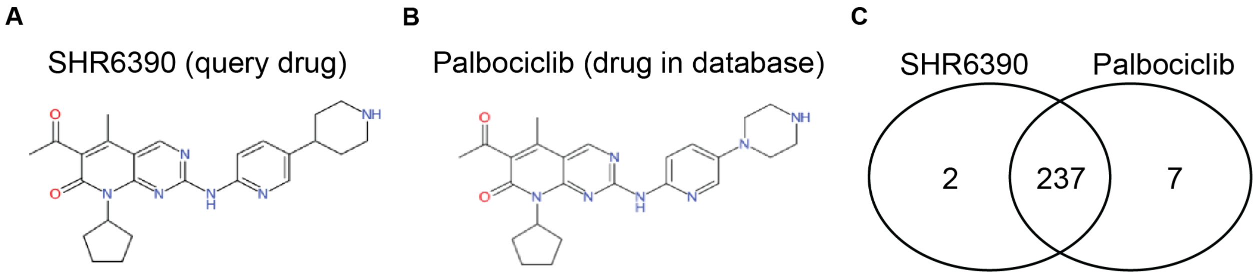

Supplementary Fig. S6.

Query results of an investigational CDK4/6 inhibitor, SHR6390, using its canonical SMILES code: CC1=C(C(=O)N(C2=NC(=NC=C12)NC3=NC=C(C=C3)C4CCNCC4)C5CCCC5)C(=O)C.

(A) 2D structure of SHR6390 generated by DepLink. **(B)** 2D structure of the top similar drug, palbociclib, which is an approved CDK4/6 inhibitor. **(C)** Comparison of biochemical features (PubChem and MACCS features; 1,047 bits in total) present in the two drugs.

Supplementary References

Behan, F.M., *et al.* Prioritization of cancer therapeutic targets using CRISPR-Cas9 screens. *Nature* 2019;568(7753):511-516.

Corsello, S.M., *et al.* Discovering the anticancer potential of non-oncology drugs by systematic viability profiling. *Nature cancer* 2020;1(2):235-248.

Dempster, J.M., *et al.* Agreement between two large pan-cancer CRISPR-Cas9 gene dependency data sets. *Nature communications* 2019;10(1):5817.

Dharia, N.V., *et al.* A first-generation pediatric cancer dependency map. *Nat Genet* 2021;53(4):529-538.

Guha, R. Chemical informatics functionality in R. *Journal of Statistical Software* 2007;18:1-16.

Lavoie, J.N., *et al.* Cyclin D1 expression is regulated positively by the p42/p44MAPK and negatively by the p38/HOGMAPK pathway. *Journal of Biological Chemistry* 1996;271(34):20608-20616.

Meyerson, M. and Harlow, E. Identification of G1 kinase activity for cdk6, a novel cyclin D partner. *Molecular and cellular biology* 1994;14(3):2077-2086.

Nassar, K.W., *et al.* Targeting CDK4/6 Represents a Therapeutic Vulnerability in Acquired BRAF/MEK Inhibitor–Resistant Melanoma. *Molecular Cancer Therapeutics* 2021;20(10):2049-2060.

Scheiblecker, L., Kollmann, K. and Sexl, V. CDK4/6 and MAPK—Crosstalk as Opportunity for Cancer Treatment. *Pharmaceuticals* 2020;13(12):418.
